# Coronal pulpotomy in mature permanent mandibular molars with irreversible pulpitis using fast setting calcium silicate cement: a case series with 1-year follow-up

**DOI:** 10.2340/biid.v12.44850

**Published:** 2025-10-15

**Authors:** Vaidehi Joshi, Roopa Babannavar, T.N Nandini, Sophia Thakur, Mallikarjun Goud

**Affiliations:** Bapuji Dental College and Hospital – Conservative Dentistry and Endodontics, Davangere, Karnataka, India

**Keywords:** mature permanent molars, pulpotomy, symptomatic irreversible pulpitis, Ultrafast Protooth

## Abstract

**Context:**

Ultrafast Protooth is a calcium silicate cement with a rapid initial setting time of 2 minutes. Its fluoride release, apatite-forming capability and favourable biocompatibility make it a potential candidate for vital pulp therapy.

**Aim:**

This case series evaluates the coronal pulpotomy outcomes using Ultrafast Protooth in permanent molars having closed apices diagnosed with symptomatic irreversible pulpitis.

**Materials and methods:**

After anaesthetisation, the tooth was isolated and disinfected, and caries were excavated. Vitality was re-assessed intra-operatively based on the bleeding response. Coronal pulpotomy was performed using Ultrafast Protooth if haemostasis was achieved within the clinically accepted time. After confirming that the material had set, based on the manufacturer’s guidelines and clinical judgement, a permanent composite restoration was placed, followed by postoperative radiographs.

**Results:**

All teeth remained asymptomatic, showing no clinical signs of pain, inflammation, or infection and responded positively to pulp sensibility tests. Radiographs confirmed no periapical disease during the 1-year follow-up.

**Conclusion:**

Ultrafast Protooth showed favourable 1-year clinical and radiographic outcomes. Continued follow-up and further controlled trials are warranted to validate these findings.

## Introduction

Dental practice has witnessed a paradigm shift towards minimally invasive approaches, with a strong emphasis on preserving the natural dentition through minimal intervention while still achieving predictable treatment outcomes [1]. The diagnosis of irreversible pulpitis in permanent teeth has traditionally implied that the dental pulp is irreversibly damaged and beyond repair, necessitating root canal treatment [2]. However, with a deeper understanding of the pulpal-periapical pathobiology and a growing emphasis on conservation, current treatment philosophies have shifted from the complete removal of pulp tissue to strategies aimed at preserving pulp vitality whenever feasible. This shift reflects a move towards biological interventions that selectively eliminate only the diseased or infected portion of the pulp, thereby supporting the body’s natural healing processes [3]. Vital pulp therapy (VPT) has become the cornerstone of modern endodontics, transforming the once-doomed exposed pulp into a tissue capable of repair and regeneration. Also, recent breakthroughs in biocompatible, anti-inflammatory and osteoinductive biomaterials have reshaped the practice of VPT [4, 5].

Mineral Trioxide Aggregate (MTA) and Biodentine are the commonly used materials for VPT. However, due to its long setting time, MTA can be washed away if not properly protected during placement [6], whereas Biodentine offers a comparatively shorter, yet debated, setting time [7]. Nevertheless, ongoing research continues to explore newer biomaterials to overcome these limitations. According to a systematic review and meta-analysis of randomised controlled trials, pulpotomies on mature permanent dentition with irreversible pulpitis in conjunction with the use of calcium silicate-based cements have shown a success rate of 92.9% [8].

Ultrafast Protooth, a calcium silicate-based cement, has been developed with VPT being among its proposed clinical applications. It is a fast-setting calcium silicate cement with a rapid initial setting time of 2 minutes. It has shown cytotoxicity similar to that of MTA and Biodentine [9]. Its calcium-silicate-aluminate formulation consists of CaO (60–70%), SiO₂ (20–30%), Al₂O₃ (< 5%), tricalcium aluminate (> 7%) and SO₄ (< 3%). Additional constituents include 3.5% fluoride by weight, nanosilica, PO₄, and 10% zirconium oxide as a radiopacifier [10]. The hydration liquid contains 2% long-chained polycarboxylic acid diluted in water. Ultrafast Protooth exhibits improved early diametral tensile strength compared to ProRoot MTA and Biodentine [11]. It has also demonstrated apatite-forming capabilities, with the deposited apatite layer shown to thicken progressively over time [12].

Ultrafast Protooth has also shown acceptable clinical performance for direct pulp capping in primary molars as well as for pulpotomy in immature permanent molars [13, 14]. However, to the best of our knowledge, no study has yet evaluated its use for coronal pulpotomy in mature permanent molars (with closed apex) diagnosed with symptomatic irreversible pulpitis.

Thus, owing to its properties and based on the available clinical evidence, the aim of this case series was to provide preliminary evidence on the use of Ultrafast Protooth for coronal pulpotomy in mature permanent molars. These findings are intended to strengthen the rationale for conducting well-designed randomised clinical trials to further validate its efficacy and long-term outcomes.

## Case selection and methodology

Clinically, permanent molars with closed apices having occlusal caries, diagnosed with symptomatic irreversible pulpitis with symptomatic apical periodontitis were selected for the series. Radiographically, caries extending ≥ 2/3 into dentine or approaching/overlapping the pulp, with a periapical index (PAI) score of 3 or lower, was used as the cutoff. The diagnosis was made based on a combined interpretation of patient symptoms, radiographic findings and a cold test performed using Endo-Frost (Roeko, Coltene). Patients included were aged between 14 and 30 years, classified as American Society of Anesthesiologists (ASA) Class I and had good oral hygiene with a healthy periodontium having probing depth of < 3 mm.

Patients were provided with comprehensive information regarding the treatment protocol, materials to be used, potential risks, follow-up schedule and alternative options. Written informed consent for participation and publication, including clinical images, was obtained from all participants in accordance with CARE guidelines.

Local anaesthesia (Lignox 2% A, Indoco) was achieved using 2% lignocaine combined with 1:80,000 adrenaline. Following rubber dam isolation, the operative site was disinfected with 5.25% sodium hypochlorite (NaOCl) (HypoChlor Forte, SafeEndo) prior to caries removal. Caries excavation was carried out using a sterile round diamond bur. Once pulpal exposure was confirmed, the coronal pulp tissue was removed up to the level of the canal orifices using a sterile round diamond bur on a high-speed handpiece.

Bleeding from each canal orifice was verified, and haemostasis was achieved by applying gentle pressure with a cotton pellet soaked in 3.25% NaOCl (HypoChlor Forte, SafeEndo) for 2 minutes, repeating the process as needed. Ultrafast Protooth was used as the pulp capping material. The material was prepared by hand-mixing to a putty-like consistency and applied to achieve a thickness of 3–4 mm over the pulp stump using a plastic-filling instrument.

Once the material had set, the total etch technique (15 seconds – enamel, 3 seconds – dentine and cement) was performed using 37% phosphoric acid (Scotchbond etchant, 3M ESPE). A universal bonding (Single bond Universal, 3M) agent was applied evenly across the surface in a scrubbing motion for 20 seconds. The adhesive was then lightly air dried for approximately 5 seconds to evaporate the solvent, followed by light curing for 10 seconds. A thin layer of flowable composite (Filtek Supreme Flowable, 3M ESPE) was applied over the pulpotomy material and light cured for 20 seconds, followed by placement of the permanent composite restoration (Filtek Z350 XT, 3M ESPE). In teeth with extensive loss of dentine structure or unsupported cusps, additional reinforcement was provided using a glass-fibre strip (Interlig, Angelus). An immediate postoperative periapical radiograph was taken to assess the outcome. All procedures were performed under 3X magnification. Table 1 represents the technical profile of the materials used in the case series.

**Table 1:** Technical profile of materials used.

Material	Composition
Ultrafast Protooth (Dentosolve, Denmark)	Powder – CaO (60–70%), SiO₂ (20–30%), Al₂O₃ (< 5%), tricalcium aluminate (> 7%) and SO₄ (< 3%). Additional constituents include 3.5% fluoride by weight, nanosilica, PO₄ and 10% zirconium oxide as a radiopacifier.Hydration liquid – 2% long-chained polycarboxylic acid diluted in water.
Scotchbond Multipurpose Etchant (3M ESPE)	37% orthophosphoric acid
Single Bond Universal Adhesive (3M ESPE)	10 MDP phosphate monomer, vitrebond copolymer, HEMA, dimethacrylate resins, fillers, silane, initiators, ethanol, water
Filtek^TM^ Supreme Flowable Restorative (3M ESPE)	Matrix: BisGMA, UDMA, bisEMA and procrylate resinsFillers: YbF_3_, nanosilica particles, nanozirconia/silica clusters
Filtek Z350 XT (3M ESPE)	Matrix – Bis-GMA, UDMA, TEGDMA, Bis-EMA, PEGDMA (partial substitution for TEGDMA to reduce shrinkage)Inorganic fillers – non-agglomerated/non-aggregated 20 nm silica. Non-agglomerated/non-aggregated 4–11 nm zirconia, Aggregated zirconia/silica cluster filler (20 nm silica + 4–11 nm zirconia)

CaO: calcium oxide; SiO₂: silicon dioxide; Al₂O: aluminium oxide; SO₄: sulphate; PO₄: phosphate; BisGMA: bisphenol A-glycidyl methacrylate; UDMA: urethane dimethacrylate; HEMA: 2-hydroxyethyl methacrylate; BisEMA: bisphenol A ethoxylated dimethacrylate; μ: microns.

**Figure 1:**
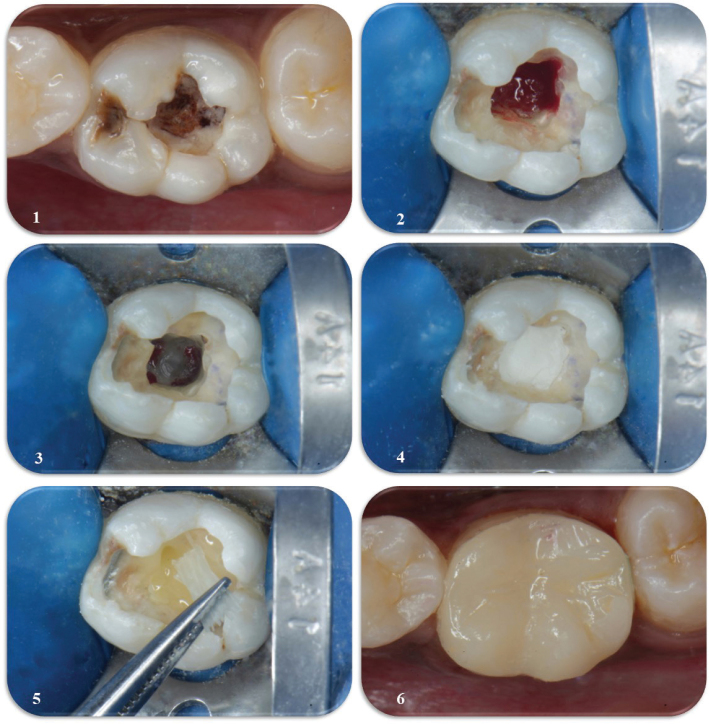
Workflow of the clinical procedures: (1) pre-operative photograph, (2) intra-operative confirmation of pulp vitality, (3) achievement of haemostasis, (4) placement of Ultrafast Protooth, (5) placement of a glass-fibre strip and (6) indirect restoration at the 6-month follow-up.

Postoperative instructions were provided, advising patients to take analgesics if needed and to report back if the pain became intolerable. A follow-up call was made 1 week after the procedure to record the status of the treated teeth. Subsequent follow-ups were scheduled at 3 months, 6 months and 1 year and 4 years for clinical and radiographic evaluations. The outcomes assessed included clinical evaluation for the presence of symptoms such as pain or sensitivity, the development of any clinical signs such as swelling or sinus tract, periodontal status of the teeth, response to pulp sensibility testing using electric pulp test (EPT) (Parkell), cold test (Endo-Frost, Roeko, Coltene) and radiographic assessment for periapical changes. Figure 1 represents the workflow of clinical procedures.

## Follow-up findings

**Figure 2:**
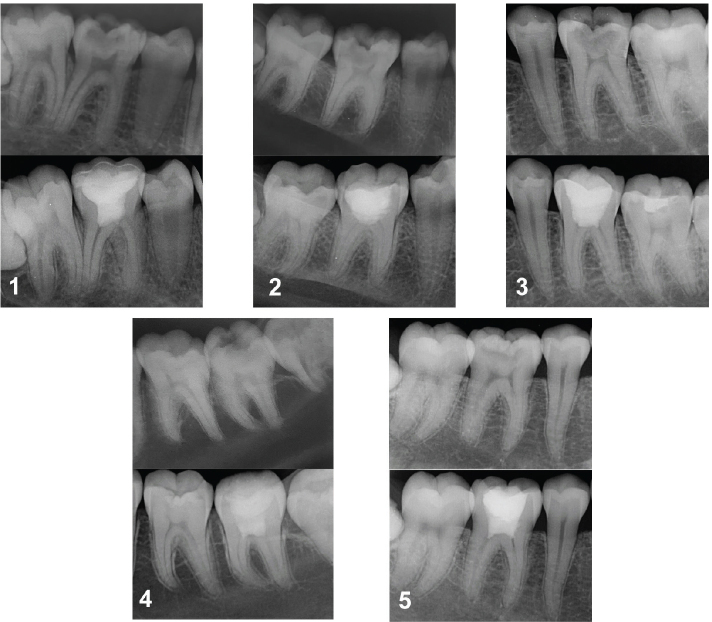
Preoperative and 1-year follow-up intra-oral periapical radiographs of cases 1–5.

During follow-up, all teeth remained asymptomatic at all times, showing no clinical signs of pain, inflammation or infection and responded positively to EPT and cold test at 1-year follow-up. Periodontal probing depth remained < 3 mm. Radiographs confirmed no periapical disease in cases 1, 4, 5 and reduction in PAI score in cases 2 and 3, underscoring the treatment’s success. The quality of the restorations was intact. Indirect restorations were placed after 6 months in cases with extensive loss of dentinal structure. A summary of case details has been mentioned in Table 2. One-year follow-up radiographs of all the cases have been provided in Figure 2.

**Table 2:** Summary of case details.

Sr. No.	Age/sex	Chief complaint	Clinical findings	Radiographic findings	Diagnosis	Follow-up findings
1.	17 years/Male	Continuous pain in the right posterior mandibular region since 3 days.	Advanced occlusal caries and tenderness on percussion with respect to tooth 46.Cold test – Lingering nature of pain	R/L* involving enamel, dentine and approaching the pulp occlusally with a PAI – 1.	Symptomatic irreversible pulpitis with symptomatic apical periodontitis	No signs/symptoms at all timesAt 1 year: EPT value 28, +ve^†^ response to cold test,No periapical changes
2.	19 years/Female	Decayed tooth and pain on chewing in relation to 46 since 1 month. Lingering in nature and aggravated upon lying down.	Advanced carious lesion involving the occlusal surface and tenderness on percussionCold test – Lingering nature of pain	R/L* involving enamel, dentine and approaching the pulp occlusallyPAI score – 2	Symptomatic irreversible pulpitis with symptomatic apical periodontitis	No signs/symptoms at all timesAt 1 year: EPT value 13, +ve response to cold test,PAI score – 1
3.	22 years/Male	Decayed tooth and pain upon hot and cold stimulus in relation to tooth 36 since 1 month. Lingering in nature.	Advanced carious lesion involving the occlusal surface and tenderness on percussionCold test – Lingering nature of pain	R/L* involving enamel, dentine and approaching the pulp occlusallyPAI score – 2	Symptomatic irreversible pulpitis with symptomatic apical periodontitis	No signs/symptoms at all timesAt 1 year: EPT value 20, +ve response to cold test,PAI score – 1
4.	15 years/Female	Continuous pain in the left posterior mandibular region since 7–8 days.	Advanced occlusal caries and tenderness on percussion with respect to tooth 37.	R/L* involving enamel, dentine and approaching the pulp occlusallyPAI score – 1	Symptomatic irreversible pulpitis with symptomatic apical periodontitis	No signs/symptoms at all timesAt 1 year: EPT value 22, +ve response to cold test,No periapical changes
5	28 years/Female	Pain in relation to tooth 46 since 15–20 days. Spontaneous onset. Lingering in nature. Aggravated in the lying down position	Advanced carious lesion involving the occlusal surface. Tooth tender on percussion.Cold test – Lingering nature of pain.	R/L* involving enamel, dentine and approaching the pulp occlusally with a periapical score of 1.	Symptomatic irreversible pulpitis with symptomatic apical periodontitis	No signs/symptoms at all timesAt 1 year: EPT value 16, +ve response to cold test,No periapical changes

EPT: electric pulp test; PAI: periapical index.

* Radiolucency;

† Positive.

## Discussion

Recent insights into pulpal pathology suggest that infection and inflammation within the pulp tend to remain compartmentalised, rather than spreading uniformly throughout the tissue. This relatively new understanding indicates that even in cases of advanced inflammation, certain regions of the pulp may remain unaffected and viable. If effective disinfection is achieved before complete necrosis sets in, these remaining vital areas may still be capable of healing and maintaining functionality [15]. In parallel, the advent of bioactive calcium silicate materials coupled with improvements in clinical techniques has enabled coronal pulpotomy as a viable, less invasive and more cost-effective alternative to root canal therapy in mature permanent teeth even with irreversible pulpitis [8, 16].

The favourable clinical and radiographic outcomes observed in this case series may be attributed, in part, to the properties of Ultrafast Protooth. Ion release from Protooth may promote apatite formation supporting the mineralisation or remineralisation of tooth structure while also potentially enhancing bonding to dentine [12]. Along with the bioactive material, a good case selection and strict adherence to antibacterial load reduction is of utmost importance in any successful VPT [17]. Pulpal health was confirmed intraoperatively by the presence of vital tissue after exposure. In all cases, haemostasis occurred within 10 min, which suggested the presence of mild to moderately inflamed pulp, which can heal in a conducive environment and is considered as an additional diagnostic as well as a prognostic indicator. Sodium hypochlorite concentrations ranging from 0.5 to 5.25% have been advocated as clinically acceptable by the European Society of Endodontology [18]. A concentration of 3.25% was used, as it provides effective disinfection while minimising the potential adverse risks [19]. The procedures meticulously adhered to the guidelines given by European Society of Endodontology and Indian Endodontic Society to avoid any confounding variables [5, 18].

The subsequent restorative procedure after the pulpotomy influences the dentine bridge formation and pulpal regeneration or repair, which in turn determines its overall success. Thus, adequate bond strength between these materials is important. A well-bonded adhesive joint between the restoration and the calcium silicate cement is advantageous, as it can evenly distribute stresses over bonded interface [20]. The surface of set Ultrafast Protooth was etched using 37% phosphoric acid, as our study (unpublished observations) on shear bond strength with different adhesive strategies found this technique to provide superior bond strength when bonded with bulk-fill flowable composite. A definitive coronal restoration was immediately placed to minimise microleakage, safeguard the bioactive medicament, reduce post-operative sensitivity and provide a stable foundation for any potential future cuspal coverage restoration [17].

In addition to procedural considerations, it is important to acknowledge some inherent limitations of coronal pulpotomy as a treatment modality. One consideration is the unpredictability of pulp sensibility testing after coronal pulpotomy. According to the European Society of Endodontology’s position statement on the management of deep caries and exposed pulp, teeth that have undergone full pulpotomy are often expected to be unresponsive to pulp sensibility testing during follow-up evaluations [18]. However, all the cases responded to EPT as well as cold test at 1 year follow-up. This is in accordance with a retrospective study performed by Arvind A. et al where 94.7% of teeth that underwent MTA pulpotomy responded to EPT at 1 year of follow-up [21]. Another potential complication is pulpal canal obliteration, which may complicate any future endodontic intervention. However, it is important to note that canal obliteration generally indicates a healthy pulp, and the likelihood of future pulp necrosis is low [22].

A key strength of this case series is the standardisation of clinical procedures, including case selection, anaesthesia, isolation, caries removal, pulp exposure management and restoration placement, which helps reduce variability and improves the reliability of outcomes. That being said, there a few limitations. First, the current follow-up is limited to 1 year. In accordance with the European Society of Endodontology (ESE) and Indian Endodontic Society (IES) guidelines, all cases will continue to be monitored for up to 4 years to evaluate the long-term clinical and radiographic outcomes. Second, assessment of dentinal bridge formation was not feasible due to the short follow-up period and the absence of baseline Cone Beam Computed Tomography (CBCT) imaging. Third, as this is a case series, the study design inherently does not include a control group. While this limits the ability to make definitive comparative conclusions regarding the biologic efficacy of Ultrafast Protooth, case series are valuable for providing preliminary clinical observations and identifying trends that can inform future controlled studies.

## Conclusion

The advancements in bioactive materials and clinical techniques are challenging the traditional diagnosis of irreversible pulpitis. Ultrafast Protooth demonstrated favourable 1-year clinical and radiographic outcomes. With this preliminary case series, Ultrafast Protooth, owing to its favourable properties, may be considered as a promising material for VPT in mature permanent teeth with symptomatic irreversible pulpitis. Its short setting time also facilitates immediate placement of permanent restorations following pulpotomy. Continued follow-up and controlled clinical trials are needed to further validate these outcomes.

## Data Availability

No additional data are available. All relevant information is contained within the case report.
